# Omental Cake: A Radiological Diagnostic Sign

**Published:** 2011-11-27

**Authors:** Naima Zamir, Jamshed Akhtar, Soofia Ahmed

**Affiliations:** Department of Paediatric Surgery National Institute of Child Health, Rafiqui Shaheed Road Karachi 75510, Pakistan

A ten-year old male child, weighing 20 kg, was admitted through emergency with abdominal fullness and pain. There was a history of sustaining a trivial injury to abdomen two months back following which he developed mild abdominal pain. Intensity of pain increased gradually and became unbearable, along with abdominal distention. There was single episode of bleeding per rectum and off and on fever during this period with no other associated symptoms like vomiting and constipation. Past medical and family history was unremarkable.

Examination showed pale, anxious, thin built child with heart rate 120/min, respiratory rate 24 breaths/min, and temperature 101° F. Abdomen was protuberant, firm and severely tender. Digital rectal examination revealed a firm mass palpable on anterior aspect of rectal wall with mobile overlying mucosa, finger stall stained with blood.

Despite the history of trauma, signs and symptoms were more in favor of abdominal tuberculosis, with the differential of post traumatic infected haematoma, and sub acute or delayed presentation of infections like appendicitis and enteric fever. His haemoglobin was 7.8 g/dl, total leukocytes 14200/cmm, neutrophils 83%, ESR 10mm in 1st hour. Ultrasound abdomen showed moderate amount of fluid in abdomino-pelvic cavity. CT scan showed thick omentum, matted gut loops with lymphadenopathy and fluid collection in the peritoneal cavity (Fig. 1, 2). 

**Figure:**
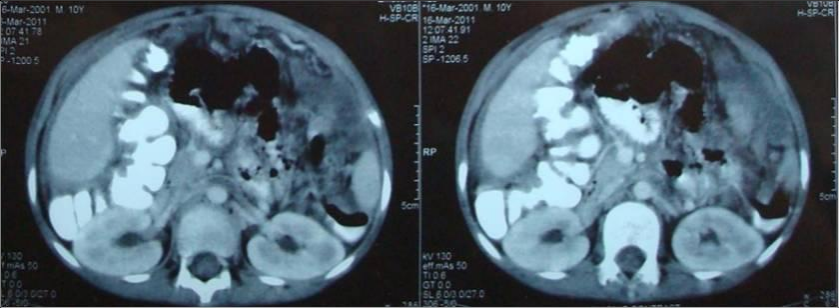
Figure 1: Thick omentum, matted gut loops with ascitic fluid collection.

**Figure:**
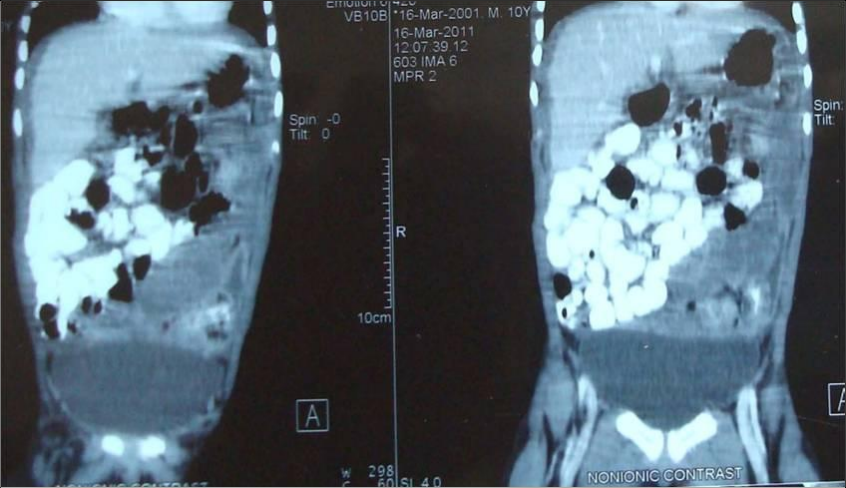
Figure 2 : Thick solid (cake) omentum pushing all the small bowel loops towards right lower quadrant with encapsulation of the colon.

## DISCUSSION

Omental spread of diseases ranges from inflammation, infection, to malignancy. Malignancy can be either primary tumor of omentum, or spread from the adjacent or remote areas. The different patterns of omental involvement include seedlings, localized or diffuse infiltration of omental fat by soft tissue density material. The resulting thickened solid omentum is called as omental caking [1].

Thick caking is an indicator of advanced stage of any disease. It is often documented in adult population. Metastatic peritoneal tumors most often originate from the ovary, stomach, pancreas, colon, uterus, and bladder. Haematogenous metastases from malignant melanoma, as well as breast and lung carcinoma, are also common. In developing countries it is also found to be associated with fibrotic type of peritoneal tuberculosis. In paediatric population it is uncommon though reported in cases of rhabdomyosarcoma and lymphoma. Mucinous adenocarcinoma of colon itself is a rare entity in paediatric population, and has an aggressive course, but omental caking is infrequently reported with this type of cancer [2, 3, 4, 5, 6, 7]. 

Computed tomography is a good diagnostic modality in cases of omental pathologies. In the normal anatomy, omentum looks like bands of fatty tissue with some fine blood vessels while in cases of ascites it appears as thin fatty layer. Soft deposits can appear as seeding. Omental caking is actually a radiological sign which on CT scan can be easily identified as diffuse haziness, or a mass like effect. The ascitic fluid encased in the thickened omentum appears as cystic spaces [3].

In our patient CT scan showed, thickened omentum almost occupying the whole of abdomen pushing the matted thick walled gut loops towards one side with ascites. These findings are very much suggestive of advanced pathology especially secondary to malignancy but since such pathology hardly documented in cases of paediatric population our provisional diagnosis was tuberculosis. CT scan findings along with the relevant clinical and demographic data can be of great help in making a diagnosis and also in planning the management of the patients with omental pathology. 

## Footnotes

**Source of Support:** Nil

**Conflict of Interest:** None declared
